# Assessment of plasma B7-H3 levels in pediatric patients with different degrees of surgical stress

**DOI:** 10.1186/s12887-016-0655-1

**Published:** 2016-07-26

**Authors:** Yan Li, Qing Yuan, Jie Huang, Yi Ping Li, Jian Pan, Xing Feng, Xue Guang Zhang, Jiang Huai Wang, Jian Wang

**Affiliations:** 1 Department of Pediatric Surgery, Affiliated Children’s Hospital, Soochow University, Suzhou, 215003 China; 2 Department of Anesthesiology, Affiliated Children’s Hospital, Soochow University, Suzhou, 215003 China; 3 Department of Pediatric Cardiology, Affiliated Children’s Hospital, Soochow University, Suzhou, 215003 China; 4 Institute of Pediatric Research, Affiliated Children’s Hospital, Soochow University, Suzhou, 215003 China; 5 Department of Neonatology, Affiliated Children’s Hospital, Soochow University, Suzhou, 215003 China; 6 Institute of Medical Biotechnology, Soochow University, Suzhou, 215006 China; 7 Department of Academic Surgery, University College Cork, Cork University Hospital, Cork, Ireland

**Keywords:** Pediatric surgery, B7-H3, Surgical stress, Children

## Abstract

**Background:**

Surgical stress initiates a series of host hormone, metabolism and immune responses, which predominantly affect the homeostatic mechanism of patients with major surgery. B7-H3 is a co-stimulatory molecule and has been shown to participate in both adaptive and innate immune responses. In this study we evaluated the clinical significance of plasma B7-H3 levels in pediatric patients with different types of operation and degrees of surgical stress.

**Methods:**

A total of 48 children received pediatric general and cardiac surgery were recruited into this study. Based on the surgical stress scoring, children were divided into moderate stress (*n* = 14) and severe stress (*n* = 34) groups. Plasma B7-H3 levels were assessed at selected time points: before surgery, immediately after surgery, at day 1, day 3, and day 7 after surgery. Correlations between plasma B7-H3 levels and surgical stress scores were also examined.

**Results:**

Plasma B7-H3 levels were significantly decreased in all 48 pediatric patients after surgery compared to the B7-H3 level before surgery (*p* < 0.01). Children with general surgery showed significant decreases in plasma B7-H3 immediately after surgery, and at day 3 and day 7 after surgery (*p* < 0.05, *p* < 0.01), whereas children with cardiac surgery showed reduced plasma B7-H3 immediately after surgery and at day 3 after surgery (*p* < 0.05). Plasma B7-H3 in cardiac surgery group was dropped much lower than that in general surgery group at day 1 (*p* < 0.05) and day 3 (*p* < 0.01) after surgery. Significantly reduced plasma B7-H3 was observed in the severe stress group, but not in the moderate stress group, immediately after surgery and at day 3 after surgery (*p* < 0.05), and severe stress group had significantly lower plasma B7-H3 levels than moderate stress group at day 1, day 3, and day 7 after surgery (*p* < 0.05). Furthermore, plasma B7-H3 levels at day 1 (*p* = 0.01) and day 3 (*p* = 0.025) after surgery correlated negatively with surgical stress scores.

**Conclusions:**

Plasma B7-H3 levels were decreased significantly in children subjected to pediatric general and cardiac surgery, which is closely associated with the severity of surgical stress. The negative correlation of plasma B7-H3 levels at day 1 and day 3 after surgery with surgical stress scoring implicates that the plasma B7-H3 level might be a useful biomarker for monitoring stress intensity during pediatric surgery.

## Background

Surgical stress initiates a series of host hormone, metabolism and immune responses, which predominantly affect the homeostatic mechanism of patients with major surgery. It has long been demonstrated for the past several decades that surgical procedures can activate the hypothalamic–pituitary–adrenal (HPA) axis, thereby causing an elevated systemic glucocorticoid level [1]. Recent studies further show markedly altered levels of multiple cytokines after surgery, and these cytokines are mainly involved in initiation of the acute inflammatory reaction and modulation of immune responses [2]. Among them, IL-6 functions as a representative proinflammatory mediator to upregulate the acute inflammatory reaction, whereas IL-10 serves as a typical anti-inflammatory cytokine to inhibit the proinflammatory response. Upregulation of these cytokines in response to surgical stress for a new balance between proinflammatory and anti-inflammatory cytokines is crucial for maintaining the homeostatic stability of the host. Nevertheless, the imbalance between these cytokines could lead to systemic inflammatory response syndrome (SIRS) or compensatory anti-inflammatory response syndrome (CARS), and even multiple-organ dysfunction syndrome (MODS) [2].

In addition, surgical procedures may bring in exogenous antigens. These exogenous antigens interact with antigen-specific T cells, thereby participating in the process of major histocompatibility complex-associated antigen presentation and T cell-mediated activation of the adaptive immune response. Importantly, this process also requires the participation of co-stimulatory molecules. B7-H3 is a co-stimulatory molecule and belongs to the B7 superfamily. B7-H3 possesses a contrasting role in regulating T cell-mediated immune responses by functioning as both a T cell co-stimulator and co-inhibitor [3]. In addition to a well-documented role of B7-H3 in regulation of T cell-mediated immune responses, it has been recently shown that B7-H3 is also involved in the innate immunity-associated inflammatory response by functioning as a co-stimulator to promote proinflammatory cytokine production [4]. Moreover, significantly enhanced soluble B7-H3 in the circulation of septic patients could predict the poor outcome of these patients [4].

In this study, peripheral blood samples were collected from children subjected to pediatric general and cardiac surgery, and plasma B7-H3 levels before surgery, immediately after surgery, and at day 1, day 3, and day 7 after surgery were measured. We further evaluated alterations in plasma B7-H3 levels before and after surgery with types of surgery and severities of surgical stress, and the related clinical significance.

## Methods

### Patients

A total of 48 children who were admitted into the surgical ward of Affiliated Children’s Hospital, Soochow University, Suzhou, China to undergo pediatric general surgery and cardiac surgery between June 2009 and December 2009 were recruited into this study. Among 48 cases, 20 cases underwent general surgery and 28 cases underwent cardiac surgery. Based on the Anand surgical stress scoring system [5], all children were further sub-grouped into moderate stress group (*n* = 14, score 6–10) and severe stress group (*n* = 34, score 11–20). The exclusion criteria included diagnosis of a genetic syndrome or malignant diseases, documented immunodeficiency or taken immunosuppressive drugs in the last 3 months, and refusal of consent. This study was approved by the Institutional Research Ethics Committee of Affiliated Children’s Hospital and Soochow University for clinical investigation, and the written informed consent was obtained from parents or guardians of the recruited children prior to enrollment. All experiments and procedures followed were conducted in accordance with the principles of the Declaration of Helsinki involving human subjects.

### Surgical stress scoring

The severity of surgical stress was assessed by the Anand scoring method, which is based on the amount of blood loss (score 0–3), the site and duration of surgery (score 0–2 and 0–4), the amount of tissue trauma (score 1–3), extent of visceral trauma (score 2–4), and associated stress factors including localized or generalized infection (score 1–2) and cardiopulmonary bypass (score 4) [5]. The total scores were used to classify the degree of surgical stress as minor (score 0–5), moderate (score 6–10), and severe (score 11–20) as described previously [5].

### Blood sampling

Blood samples (2.0 ml) were obtained at the following time points: before surgery (Tc), immediately after surgery (T0), at day 1 (T1), day 3 (T3), and day 7 (T7) after surgery. At each time point, freshly heparinized blood samples were collected and centrifuged. Plasma samples were harvested and stored at −80 °C until analysis.

### Measurement of plasma soluble B7-H3

An ELISA kit for soluble B7-H3 detection, with anti-B7-H3 mAbs 4H7 as a capture antibody and biolinylated 21D4 as a detecting antibody, was previously developed in our laboratory (6). In the current study, plasma concentrations of soluble B7-H3 was assessed using a modified ELISA kit in which the biolinylated 21D4 mAb was replaced by the biolinylated 2E6 mAb as a detecting antibody, with an improved sensitivity of soluble B7-H3 detecting limit from a previous 27 pg/ml to 3.3 pg/ml (4).

### Prognosis of patients

Postoperative complications including hemorrhage, fever, hypothermia, infection, and incision rupture were monitored and recorded. In addition, the length of hospital stay was also recorded.

### Statistical analysis

Statistical analysis was performed using SPSS17.0. All data were tested for normal distribution and expressed as mean ± standard deviation (SD) or otherwise presented as median (inter-quartile range). The Friedman test was used to compare the medians in multiple groups and the Spearman rank correlation coefficient test was used to analysis correlations between plasma soluble B7-H3 levels and surgical stress scores. A *p*-value of less than 0.05 was considered to be statistically significant.

## Results

### Patient information

Patient’s data and clinical characteristics are summarized in Table 1. Among 48 cases, there were 23 males and 25 females with an average age of 12.74 ± 17.69 months. The length of hospital stay was 16.48 ± 6.75 days, and white blood cell counts, neutrophil counts, and C-reactive protein concentrations before surgery were (8.79 ± 4.44) × 10^9^/L, 61.0 ± 8.82 %, and 3.26 ± 4.17 mg/L, respectively. There were more cases with cardiac surgery but less cases with general surgery in severe stress group compared to moderate stress group (*p* < 0.01). Moreover, children in the severe stress group had much longer hospital stay than children in the moderate stress group (*p* < 0.05).

**Table 1:** Patient’s data and characteristics

	Total patients	Moderate stress	Severe stress	*P* values
Male / female	23/25	9/5	14/20	0.111
Age (months)	12.7 ± 17.7	8.87 ± 19.4	13.9 ± 17.3	0.004
General surgery (cases %)	20 (41.7)	14 (100)	6 (17.6)	0.000
Cardiac surgery (cases %)	28 (58.3)	0 (0)	28 (82.4)	0.000
Total white cell counts before surgery (10^9^/L)	8.79 ± 4.44	8.87 ± 5.70	8.74 ± 3.54	0.513
PMN counts before surgery (%)	61.0 ± 8.82	60.6 ± 7.31	61.6 ± 10.8	0.124
CRP (mg/L)	3.26 ± 4.17	2.78 ± 4.25	3.26 ± 4.21	0.725
Hospital stay (days)	16.5 ± 6.75	13.4 ± 6.28	19.5 ± 7.22	0.031

*PMN* polymorphonuclear neutrophil, *CRP* C-reactive protein

All data are expressed as mean ± SD. *P* values: comparisons between moderate and severe stress groups

### Significantly reduced plasma B7-H3 levels after surgery

**Fig. 1:**
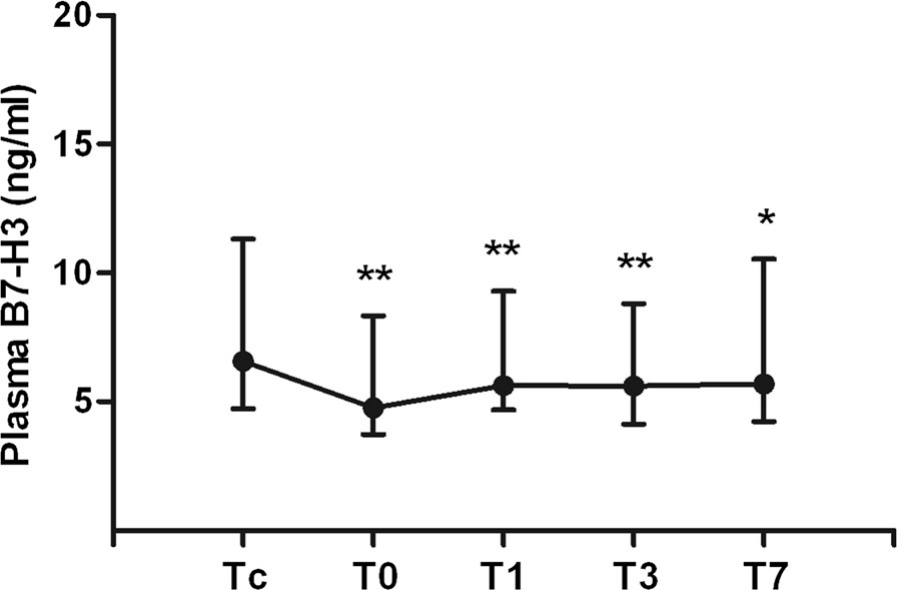
Plasma B7-H3 levels were significantly decreased after surgery. Heparinized whole blood samples were collected from 48 children subjected to pediatric general and cardiac surgery at following time points: before surgery (Tc), immediately after surgery (T0), at day 1 (T1), day 3 (T3), and day 7 (T7) after surgery. Plasma soluble B7-H3 was assessed by ELISA. Data are presented as median (inter-quartile range). *p < 0.05, **p < 0.01 compared with plasma B7-H3 before surgery (Tc)

As shown in Fig. 1, soluble B7-H3 concentrations in the circulation were significantly decreased in all 48 pediatric patients after general and cardiac surgery compared to the B7-H3 level before surgery (*p* < 0.05, *p* < 0.01). The plasma B7-H3 level before surgery was 6.58 (4.72–11.32) ng/ml; however, it dropped to 4.75 (3.73–8.34) ng/ml immediately after surgery (*p* = 0.000), 5.63 (4.68–9.29) ng/ml at day 1 (*p* = 0.009), 5.61 (4.12–8.81) ng/ml at day 3 (*p* = 0.000), and 5.61 (4.12–8.81) ng/ml at day 7 (*p* = 0.030) after surgery.

**Fig. 2:**
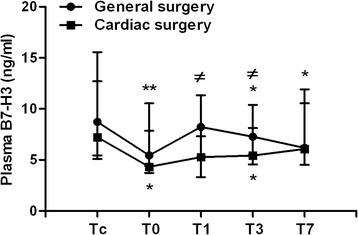
The effect of pediatric general and cardiac surgery on plasma B7-H3 levels. Heparinized whole blood samples were collected from children with general surgery (*n* = 20) and children with cardiac surgery (*n* = 28) at following time points: before surgery (Tc), immediately after surgery (T0), at day 1 (T1), day 3 (T3), and day 7 (T7) after surgery. Plasma soluble B7-H3 was assessed by ELISA. Data are presented as median (inter-quartile range). *p < 0.05, **p < 0.01 compared with plasma B7-H3 before surgery (Tc); ^≠^ *p* < 0.05 compared with cardiac surgery group

We further assessed plasma B7-H3 levels in children subjected to either general surgery or cardiac surgery. When compared to the B7-H3 level before surgery at 8.73 (5.44–15.56) ng/ml, children with general surgery had significantly decreased plasma B7-H3 immediately after surgery at 5.44 (4.40–10.54) ng/ml (*p* = 0.002), and at day 3 at 7.28 (5.53–10.39) ng/ml (*p* = 0.006) and day 7 at 6.17 (4.53–11.91) ng/ml (*p* = 0.028) after surgery, whereas children with cardiac surgery showed significantly reduced plasma B7-H3 immediately after surgery at 4.32 (3.72–7.86) ng/ml (*p* = 0.015) and at day 3 at 5.43 (3.35–8.12) ng/ml (*p* = 0.013) after surgery (Fig. 2). Furthermore, plasma B7-H3 in cardiac surgery group was dropped much lower than that in general surgery group at day 1 (*p* < 0.05) and day 3 after surgery (*p* < 0.01) (Fig. 2).

### Correlation between plasma B7-H3 and surgical stress scoring

**Fig. 3:**
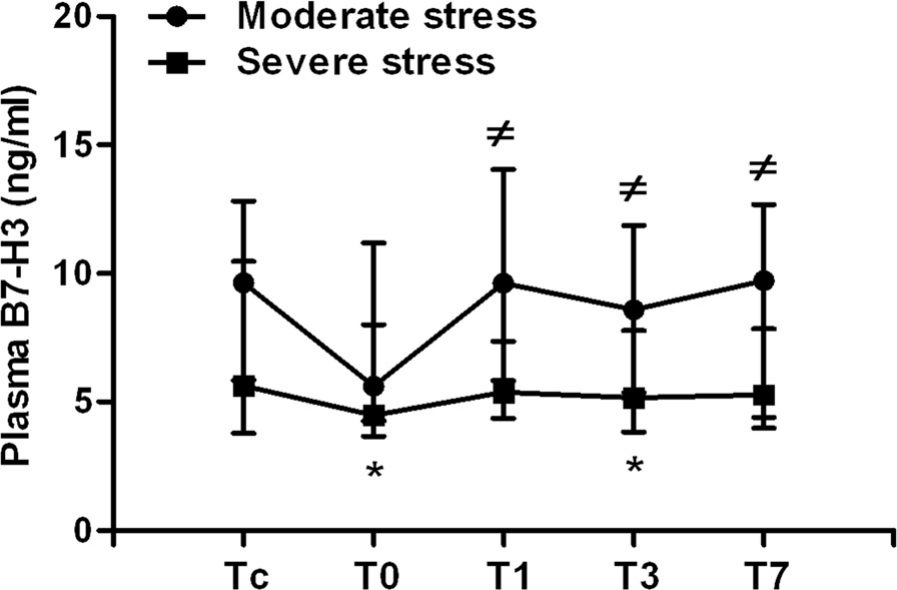
Moderate and severe surgical stresses differentially affect plasma B7-H3 levels. Heparinized whole blood samples were collected from children with moderate surgical stress (*n* = 14) and children with severe surgical stress (*n* = 34) at following time points: before surgery (Tc), immediately after surgery (T0), at day 1 (T1), day 3 (T3), and day 7 (T7) after surgery. Plasma soluble B7-H3 was assessed by ELISA. Data are presented as median (inter-quartile range). *p < 0.05 compared with plasma B7-H3 before surgery (Tc); ^≠^ *p* < 0.05 compared with severe stress group

We next analyze plasma B7-H3 levels between moderate stress group and severe stress group before and after surgery. As shown in Fig. 3, there were no significant differences in plasma B7-H3 levels observed in moderate stress group after surgery compared to the B7-H3 level before surgery; however, significantly reduced plasma B7-H3 was seen in severe stress group immediately after surgery at 4.47 (3.65–8.01) ng/ml (*p* = 0.014) and at day 3 after surgery at 5.15 (3.97–7.84) ng/ml (*p* = 0.017) compared to the plasma B7-H3 level at 5.62 (3.78–10.48) ng/ml before surgery. In addition, children in the severe stress group had significantly lower plasma B7-H3 than children in the moderate stress group at day 1, day 3, and day 7 after surgery (*p* < 0.05) (Fig. 3).

Plasma B7-H3 levels in children subjected to pediatric general and cardiac surgery correlated negatively with the Anand surgical stress scores at day 1 after surgery (R-square = −0.367, *p* = 0.010) and at day 3 after surgery (R-square = −0.324, *p* = 0.025), respectively (Table 2).

**Table 2:** Correlations of plasma B7-H3 levels with the Anand surgical stress scores at different time points before and after surgery

Plasma B7-H3	Anand surgical stress scores	
	R-square	*p* value
Before surgery	−0.218	0.136
Immediately after surgery	−0.194	0.188
At day 1 after surgery	−0.367	0.010
At day 3 after surgery	−0.324	0.025
At day 7 after surgery	−0.218	0.137

R2 values were calculated using the Spearman rank correlation coefficient test

### Correlations of surgical stress scoring with prognosis of patients

**Fig. 4:**
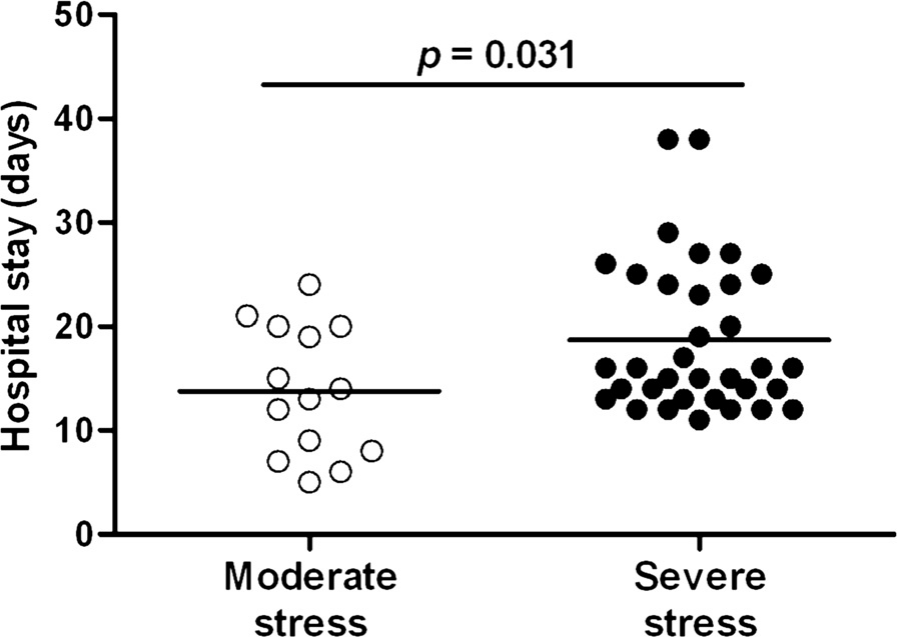
Children with severe surgical stress show significantly longer hospital stay than children with moderate surgical stress. The length of hospital stay between children in the moderate stress group (*n* = 14) and in the severe stress group (*n* = 34) was recorded and compared. *p* = 0.031 compared with the moderate stress group

As shown in Fig. 4, children in the severe stress group showed much longer hospital stay than children in the moderate stress group (*p* < 0.05). Moreover, the Anand surgical stress scores correlated positively with postoperative complications (R-square = 0.288, *p* = 0.047) (Table 3). However, plasma B7-H3 levels after surgery in all measured time points did not correlate with either the length of hospital stay or postoperative complications (data not shown).

**Table 3:** Correlation between postoperative complications and the Anand surgical stress scores

	Anand surgical stress scores	
	R-square	*p* value
Postoperative complications	0.288	0.047

The R2 value was calculated using the Spearman rank correlation coefficient test

## Discussions

Host immune system is affected and regulated by multiple mediators and factors including stress, infection, trauma, malignant diseases, and nutrient conditions. Nevertheless, major surgery also affects and modulates the host homeostasis and defense mechanisms. In the present study, we measured plasma B7-H3 levels in 48 children subjected to pediatric general and cardiac surgery at different time points before and after surgery. We found that children with general surgery showed significantly reduced plasma B7-H3 immediately after surgery, and at day 3 and day 7 after surgery, whereas children with cardiac surgery showed significantly reduced plasma B7-H3 immediately after surgery and at day 3 after surgery. Significantly reduced plasma B7-H3 immediately after surgery and at day 3 after surgery was also observed in children with severe surgical stress, but not in children with moderate surgical stress. Moreover, plasma B7-H3 levels at day 1 and day 3 after surgery correlated negatively with the surgical stress scoring. These results indicate for the first time that the altered plasma B7-H3 level after surgery is associated with the degree of surgical stress in children who undergo major surgery.

Soluble B7-H3 in the circulation is shed from membrane B7-H3 expressed on the cell surface of human monocyte, dendritic cell, and active T cells [6], where matrix metalloproteinases (MMPs) play a key role to cleave the membrane B7-H3 into soluble B7-H3 via the enzymatic digestion [7]. Circulating soluble B7-H3 is detectable but usually at very low level in normal humans; however, patients with liver cancer [8] or colon cancer [9], and children with mycoplasma pneumonia [10] presented substantially enhanced B7-H3 levels in their circulation. Wei et al. [11] reported that significantly lower B7-H3 levels in the prostatic fluid were observed in patients with chronic prostatitis compared to healthy volunteers, which correlates negatively with the patients’ disease status. In the present study, we also found that plasma soluble B7-H3 levels decreased significantly after surgery in children subjected to pediatric general and cardiac surgery. It has been well-documented that B7-H3, as a co-stimulatory molecule, participates in both adaptive and innate immune responses [3, 4]. Thus, the reduced plasma B7-H3 level after surgery may reflect the decreased and/or depressed host immune response in children with major surgery.

It is generally believed that surgery could suppress host immune responses, at least for a short time period [12–14]. However, other studies have shown that surgery does not alter or even enhance immune responses [15–17]. An early study by Mollitt et al. [18] revealed that neutrophil phagocytosis, migration, chemotaxis, and killing were not affected by surgery in children who underwent minor surgery. Interestingly, Romeo et al. [19] reported an enhanced activity of monocytes observed in children who underwent minor surgery, whereas Merry et al. [20] found no alterations in neutrophil chemotaxis and actin polymerization seen in children who underwent major surgery. These results implicate that different components of host immune system such as monocytes and neutrophils may respond differentially under different surgical stress conditions. Nevertheless, our results indicate the negative impact of major surgery on host immune responses in children.

As plasma B7-H3 levels were dropped substantially after surgery, we next examine whether the reduced plasma B7-H3 correlates with the severity of surgical stress. All 48 children subjected to pediatric general and cardiac surgery were scored using the Anand scoring method to evaluate the severity of surgical stress and sub-grouped into moderate stress group and severe stress group based on their surgical stress scores. Notably, children in the severe stress group showed significantly lower plasma B7-H3 than children in the moderate stress group at day 1, day 3, and day 7 after surgery, indicating that the plasma B7-H3 level is associated with the severity of surgical stress. Furthermore, a negative correlation was revealed between plasma B7-H3 levels at day 1 and day 3 after surgery, but not immediately after surgery and at day 7 after surgery, with surgical stress scores, indicating that surgical stress may reach peak point during the period of day 1 and day 3 after surgery. Zhao et al. [21] selectively inhibited histone deaccetylase-6 to attenuate the stress response in a murine cecal ligation and puncture (CLP)-induced sepsis model and found that the maximized stress response was at day 3 after CLP, which is in consistence with our findings. Our results also revealed that children in the severe stress group had much longer stay in the hospital than children in the moderate stress group, and the Anand surgical stress scores correlated positively with postoperative complications. However, neither the length of hospital stay nor postoperative complications correlated with the plasma B7-H3 levels before surgery, immediately after surgery, at day 1, day 3, and day 7 after surgery.

The major limitation of the current study is the relative small numbers of recruited pediatric patients, in particular when these patients were further sub-grouped into moderate and severe stress groups. Consequently, although reduced plasma B7-H3 levels after surgery correlated negatively with the surgical stress scoring and were closely associated with the severity of surgical stress, they failed to correlate with the clinical outcomes including postoperative complications and the length of hospital stay.

## Conclusions

In this study we demonstrated that plasma soluble B7-H3 in children subjected to pediatric general and cardiac surgery were significantly decreased in response to surgical stress, which correlates negatively with the Anand surgical stress scores. These results suggest the clinical significance for detection of plasma B7-H3 in the clinical setting as a biomarker to monitor the stress intensity during pediatric surgery, though large scale of studies will be needed to confirm this.

## Abbreviations

CARS, compensatory anti-inflammatory response syndrome; CLP, cecal ligation and puncture; HPA, hypothalamic–pituitary–adrenal; MMPs, matrix metalloproteinases; MODS, multiple-organ dysfunction syndrome; SIRS, systemic inflammatory response syndrome
